# Characteristics of gut microbiota in patients with asthenozoospermia: a Chinese pilot study

**DOI:** 10.1186/s12866-023-03173-5

**Published:** 2024-01-15

**Authors:** Yang Pan, Shangren Wang, Li Liu, Xiaoqiang Liu

**Affiliations:** Department of Urology, Tianjin Medical University General Hospital, 154 Anshan Road, Heping District, Tianjin, 300052 China

**Keywords:** Asthenozoospermia, Semen quality, Gut microbiota, Dysbiosis, 16S rRNA

## Abstract

**Background:**

Identification of intestinal flora composition is significant for exploring the cause and pathogenic mechanisms of the gut-testis axis and clarifying the relationship between microbiota and infertility. Our study aimed to examine the alternation in gut microbiota composition and identify potential microbes associated with development of Asthenozoospermia (AS).

**Method:**

A total of 580 males were recruited in the outpatient department of Tianjin Medical University General Hospital between September 2021 and March 2023. Sperm parameters were analyzed according to the WHO laboratory manual. The 16 S rRNA gene high-throughput sequencing was performed to detect the gut microbiota composition in fecal samples. LEfSe analysis was used to screen key microbiota. PICRUSt2 software was utilized to predict relevant pathways.

**Results:**

After rigorous screening, 60 isolated AS patients (AS group) and 48 healthy men (NC group) were enrolled. No significant differences were observed in demographic characteristics (*p* > 0.05), semen volume (*p* = 0.718), sperm concentration (*p* = 0.109), or total sperm count (*p* = 0.200). Sperm total motility and progressive motility were significantly decreased in the AS group (*p* < 0.001). AS patients had significantly lower alpha diversity indices (Chao1, observed OTUs, and PD Whole-tree; *p* < 0.05). The beta-diversity of gut microbiota in AS patients significantly differed from NC men (PCoA analysis, *p* = 0.001). *Firmicutes*, *Bacteroidota*, *Proteobacteria*, and *Actinobacteria* were the primary phyla, with the dominant genera including *Bacteroides*, *Prevotella*, and *Blautia*. Eleven key genera such as *Escherichia_Shigella* and *Prevotellaceae_UCG_001* were identified by LEfSe analysis. Most of these genera were negatively correlated with sperm mobility. Eighty-eight KEGG pathways, including steroid biosynthesis and meiosis, were significantly enriched between the two groups.

**Conclusions:**

It appears that gut microbiota composition in AS patients significantly differed from that in healthy men, and the development of AS might be associated with intestinal flora dysbiosis.

**Supplementary Information:**

The online version contains supplementary material available at 10.1186/s12866-023-03173-5.

## Introduction

Asthenozoospermia (AS) is one of the most frequent reasons for infertile men. It is characterized by a reduced sperm progressive motility to < 32%. AS is usually identified as an isolated illness or as one aspect of other semen anomalies [1]. The etiologies of AS are complex and varied, such as inflammation, immune defects, irregular lifestyles, and genetics [2].

Gut microbiota could play a role in human immune and causative agent resistance [3, 4]. Gut microbiota dysbiosis is usually associated with an abnormality in microbial diversity, resulting in inflammation and autoimmune diseases [5, 6]. Moreover, intestinal flora participates in the regulation of inflammatory and immune protection in many organs such as the brain and testes [7, 8].

Gut microbiota dysbiosis could affect the integrity of the blood-testis barrier (BTB), eventually impairing testicular spermatogenic processes by potential mechanisms below. On one hand, the testes usually cannot synthesize nutrients themselves. Blood vessels in the testes transport nutrients, including those synthesized or metabolized by the gut microbiota, from the digestive system to the testicular interstitium. These nutrients, such as vitamins and minerals, are vital for normal testicular function [9]. Gut microbiota dysbiosis may disturb the original nutritional structure and subsequently affect testicular function [10]. On the other hand, gut microbiota dysbiosis may result in a chronic inflammatory status and excessive immunological response that disrupts the spermatogenic processes in the testes [11]. For example, gut microbiota dysbiosis could cause abnormal intestinal permeability and increase lipopolysaccharide (LPS) levels in the blood. The increased LPS can induce innate immunity and activate testicular LPS/TLR4/MyD88/NF-κB pathways [12]. This process can induce testicular endothelial injury and damage the BTB, eventually impairing spermatogenesis.

Identifying intestinal flora composition is significant for understanding the causes and pathogenic mechanism of the gut-testis axis and clarifying the relationship between the microflora and infertility. There are few reports on specifically investigating the gut microbiota characteristics in isolated AS patients. Hence, our study aimd to examine the microbiota characteristics in the gut of AS patients and discover the potentially key gut microbiota associated with the development of AS.

## Materials and methods

### Study participants

**Fig. 1:**
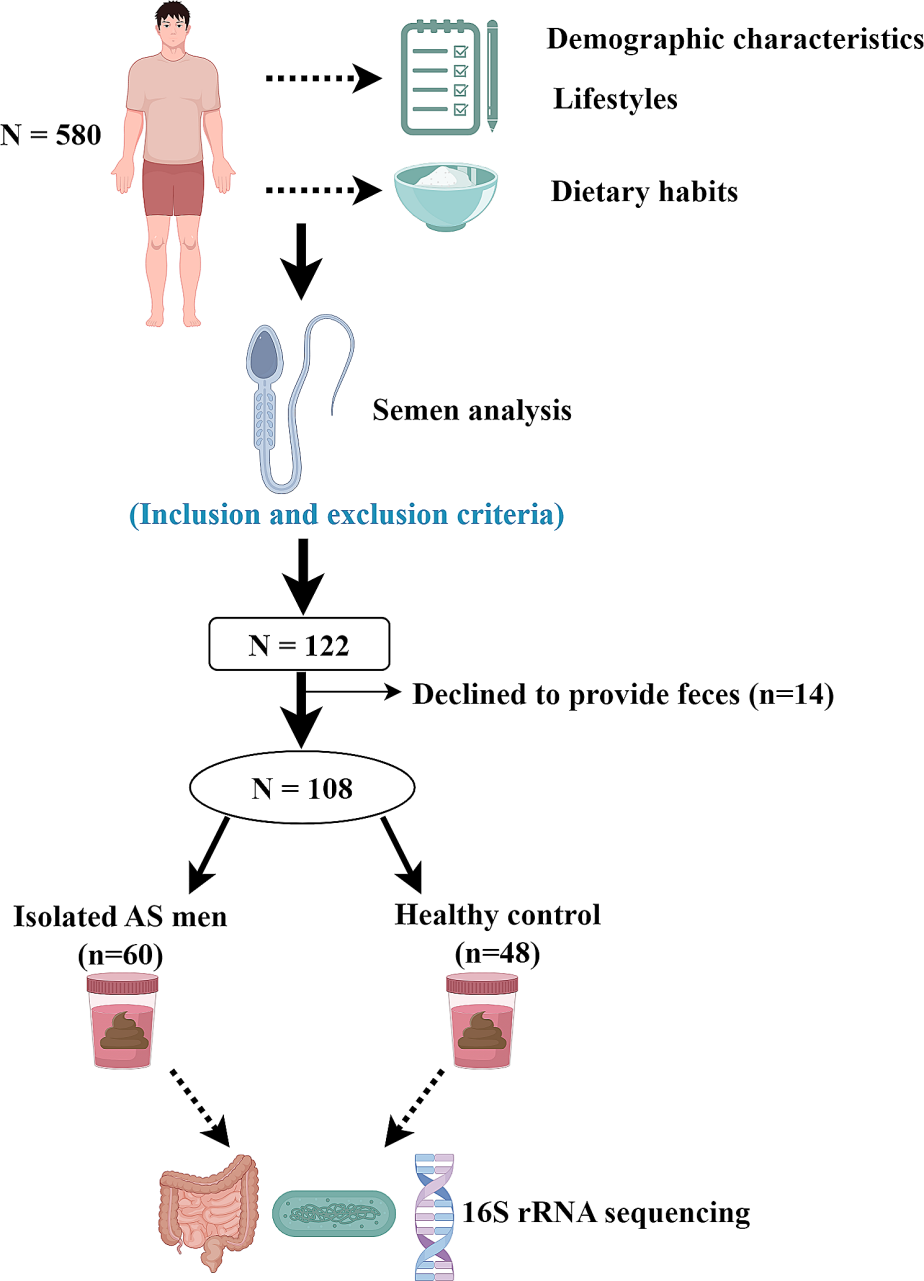
Schematic representation of the study design

A flowchart of the study design is shown in Fig. 1. Male patients were recruited in the outpatient department of Tianjin Medical University General Hospital. The study started in September 2021 and ended in March 2023. A total of 580 males were recruited during the study. Patients were diagnosed with isolated AS according to semen analysis results. Healthy men with normal semen were regarded as normal controls. Demographic characteristics and clinical parameters were recorded in detail. The inclusion criteria were as follows: (1) age between 20 and 40 years old; (2) not use antibiotics and hormone drugs in 6 months; (3) not suffer from genetic illnesses. The exclusion criteria were: (1) AS patient was not isolated AS, and combined with other semen abnormality; (2) men had chronic diseases such as hypertension, diabetes, and cardiovascular diseases; (3) men had other clinical symptoms or diseases (such as depression and inflammatory bowel disease) could potentially impact the intestinal flora outcomes; (4) men used the probiotics or prebiotics in the past six months. The study was performed according to the Declaration of Helsinki and approved by the Ethics Committee of Tianjin Medical University General Hospital (IRB2022-KY-308). Written informed consent was obtained from all participants. All data will be available from the corresponding author on reasonable request.

### Collection of semen samples

Semen was collected according to the World Health Organization (WHO) laboratory manual for the examination and processing of human semen. Abstinence was continued for 3–5 days and semen was generated by masturbation. Before collecting semen samples, the hands and penises of these males were washed using warm soapy water 3 times and then wiped with 75% alcohol. Semen was directly ejaculated into a sterile container. Sperm parameters were tested and analyzed as per the WHO laboratory manual.

### Feces specimen collection

A total of 108 fresh fecal samples were collected for gut microbiome analysis. Each man provided a single fecal sample. All fecal samples were collected using sterile and DNase-free containers and stored at − 80 °C until DNA extraction.

### DNA extraction and quality checked

Feces genomic DNA was extracted using E.Z.N.A. Stool DNA Kit (Omega Bio-tek, Inc., USA) following the manual. The concentration and quality were checked by a NanoDrop 2000 spectrophotometer (Thermo Scientific Inc., USA). DNA samples were stored at − 20 ℃ for further experiments.

### Analysis of the gut microbiota

The V3-4 hypervariable region of bacterial 16S rRNA gene were amplified with the universal primer 338F (5’-ACTCCTACGGGAGGCAGCAG-3’) and 806R (5’- GGACTACNNGGGTATCTAAT-3’). Raw data were divided into different samples according to the barcode sequence through QIIME (v1.8.0) software. A detailed analysis method was described in the supplementary file 1.

### Statistical analysis

Dichotomous variables were presented as frequencies and compared using the chi-square test. Continuous variables were presented as mean (standard deviation, SD) or median (interquartile ranges, IQR). They were compared using the independent Student’s t-test or Wilcoxon rank-sum test. Statistical analyses were conducted using SPSS 23.0 (SPSS Inc., Chicago, IL, USA) and Project R software (v3.6.0). The value of *p* < 0.05 was considered statistically significant.

## Results

### Clinical characteristics

After rigorous screening, 108 men were enrolled in this study, including 60 isolated AS men (AS group) and 48 healthy control men (NC group). Demographic characteristics of the participants are shown in Table 1. In general, no significant differences between the two groups were observed in age (*p* = 0.570), weight (*p* = 0.696), height (*p* = 0.810), and body mass index (BMI; *p* = 0.794). There were also no significant differences in lifestyles including smoking, alcohol consumption, and physical exercise (*p* > 0.05). Besides, analysis of dietary habits showed no significant differences between the two groups in tea consumption, coffee consumption, egg consumption, soy or dairy consumption, meat consumption, and vegetable consumption (*p* > 0.05). Therefore, demographic characteristics, lifestyles, and dietary habits were comparable between the AS group and the NC groups.

**Table 1:** Demographic characteristics and sperm parameters for participants

	AS group (n = 60)	NC group (n = 48)	*p*-value
Age (years) ^a^	31.55 ± 4.25	30.75 ± 4.04	0.570
Weight (kg) ^a^	73.58 ± 6.70	74.50 ± 7.33	0.696
Height (m) ^a^	1.76 ± 0.04	1.76 ± 0.07	0.810
BMI (kg/m^2^) ^a^	23.76 ± 2.10	23.95 ± 2.24	0.794
Smoking habits (n, %) ^b^			
*≥ 5 cigarettes/day*	24 (40%)	16 (33.3%)	0.476
Alcohol consumption (n, %) ^b^			
*≥ 2 times/week*	28 (46.7%)	26 (54.2%)	0.439
Tea consumption (n, %) ^b^			
*≥ 3 times/week*	12 (20%)	12 (25%)	0.535
Coffee consumption (n, %) ^b^			
*≥ 3 times/week*	18 (30%)	15 (31.3%)	0.889
Egg consumption (n, %) ^b^			
*≥ 5 times/week*	54 (90%)	45 (93.8%)	0.728
Soy or dairy consumption (n, %) ^b^			
*≥ 5 times/week*	51 (85%)	39 (81.3%)	0.603
Meat consumption (n, %) ^b^			
*≥ 100 g/day*	15 (25%)	18 (37.5%)	0.161
Vegetable consumption (n, %) ^b^			
*≥ 5 times/week*	45 (75%)	42 (87.5%)	0.103
Physical exercise (n, %) ^b^			
*≥ 2 times/week*	35 (58.3%)	25 (52.1%)	0.516
Semen volume (mL) ^c^	3.04 (2.71–5.15)	3.18 (2.25–4.86)	0.718
Total sperm count (million) ^c^	210.65 (108.82–382.2)	281.00 (189.36-394.39)	0.200
Sperm concentration (million/mL) ^c^	57.87 (28.77–87.93)	96.77 (48.72-137.81)	0.109
Total sperm mobility (%) ^c^	30.70 (23.25–34.38)	59.00 (43.44–63.73)	< 0.001
Sperm progressive mobility (%) ^c^	21.22 (14.58–26.01)	54.81 (40.17–61.39)	< 0.001

**Abbreviations**: **AS group**, Asthenozoospermia group; **NC group**, Normal control group; **BMI**, body mass index

^**a**^ Data were presented as mean and standard deviation (SD), and were compared using the independent Student’s t test

^**b**^ Data were presented as numbers and proportions and compared using the chi-square test

^**c**^ Data were presented as medians and interquartile ranges 25^th^-75^th^ percentiles (IQR), and were compared using the Mann-Whitney U test

### Sperm parameters

The median semen volume was 3.04 (IQR 2.71–5.15) mL in the AS group and 3.18 (IQR 2.25–4.86) mL in the NC group, with no significant difference (*p* = 0.718). Similarly, no significant differences between the two groups were observed in sperm concentration (*p* = 0.109) and total sperm count (*p* = 0.200). The median sperm total mobility in percentage was 30.70 (IQR 23.25–34.38) in the AS group and 59.00 (IQR 43.44–63.73) in the NC group, while the sperm progressive mobility in percentage was 21.22 (IQR 14.58–26.01) and 54.81 (IQR 40.17–61.39), respectively. Compared to those of the NC group, sperm total motility (*p* < 0.001) and progressive sperm motility (*p* < 0.001) were significantly decreased in the AS group. All sperm parameters are summarized in Table 1.

### Altered diversity of the intestinal flora

**Fig. 2:**
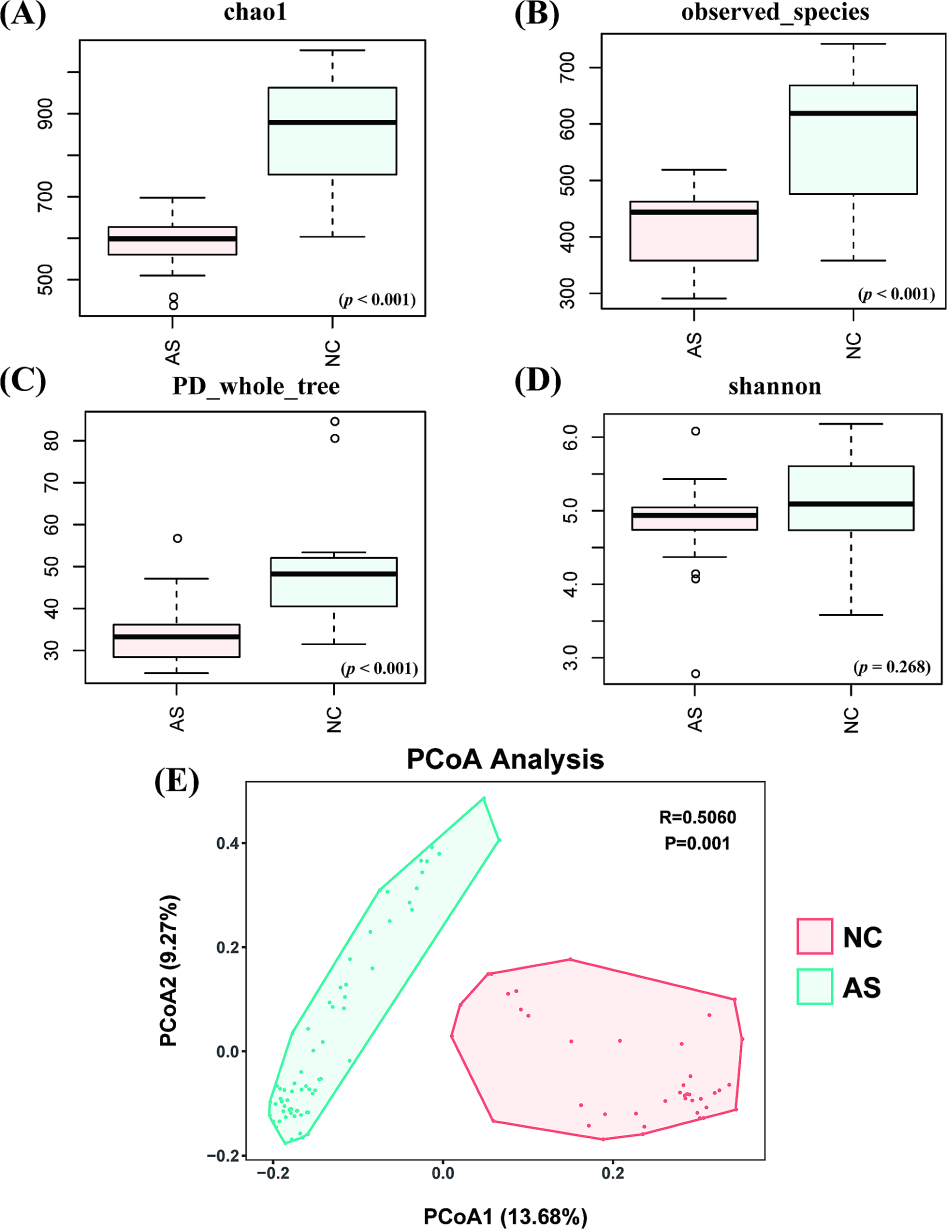
The alpha diversity indices and beta-diversity analysis between the AS group and NC group. (**A**) Chao1 index, (**B**) observed OTUs index, (**C**) PD Whole tree index, and (**D**) Shannon index between the AS group and NC group; (**E**) PCoA analysis using the unweighted UniFrac method

After sequence processing and filtering, the average read count per sample was 52,998 (range, 28,304 to 99,487). The sequence depth was visualized using a rarefaction curve. The curves for all samples were nearly horizontal with increasing sequencing depth, indicating that the depth was appropriate. The rate of increase in operational taxonomic units (OTUs) in the NC group quickly exceeded that in the AS group, indicating AS patients had a relatively lower degree of taxa abundance. Alpha diversity reflected the abundance and diversity of microbial communities. Consistently, the test depicted significantly lower species richness (Chao1 index and observed OTUs) in the AS group than in the NC group (*p* < 0.001, Fig. 2A, B). The Shannon index, which measures both richness and evenness, was not significantly different between two groups (*p* = 0.268, Fig. 2D). However, the PD Whole tree index, which also measures both richness and evenness, was significantly lower in the AS group than that in the NC group (*p* < 0.001, Fig. 2C). Therefore, there was a noticeable and significant decrease in the alpha diversity indices of AS men compared to NC men.

To evaluate extent of similarity between microbiota communities, we calculated beta-diversity values using the unweighted UniFrac method. Principal co-ordinates analysis (PCoA) illustrated that AS patients significantly differed from NCs (*p* < 0.01, Fig. 2E). Furthermore, ANOSIM (analysis of similarities) revealed significant difference between the AS and NC group (ANOSIM, R statistic = 0.506, *p* = 0.001). These results revealed a remarkable alteration in the gut microbiome between the two groups.

### Taxonomic changes of intestinal flora

**Fig. 3:**
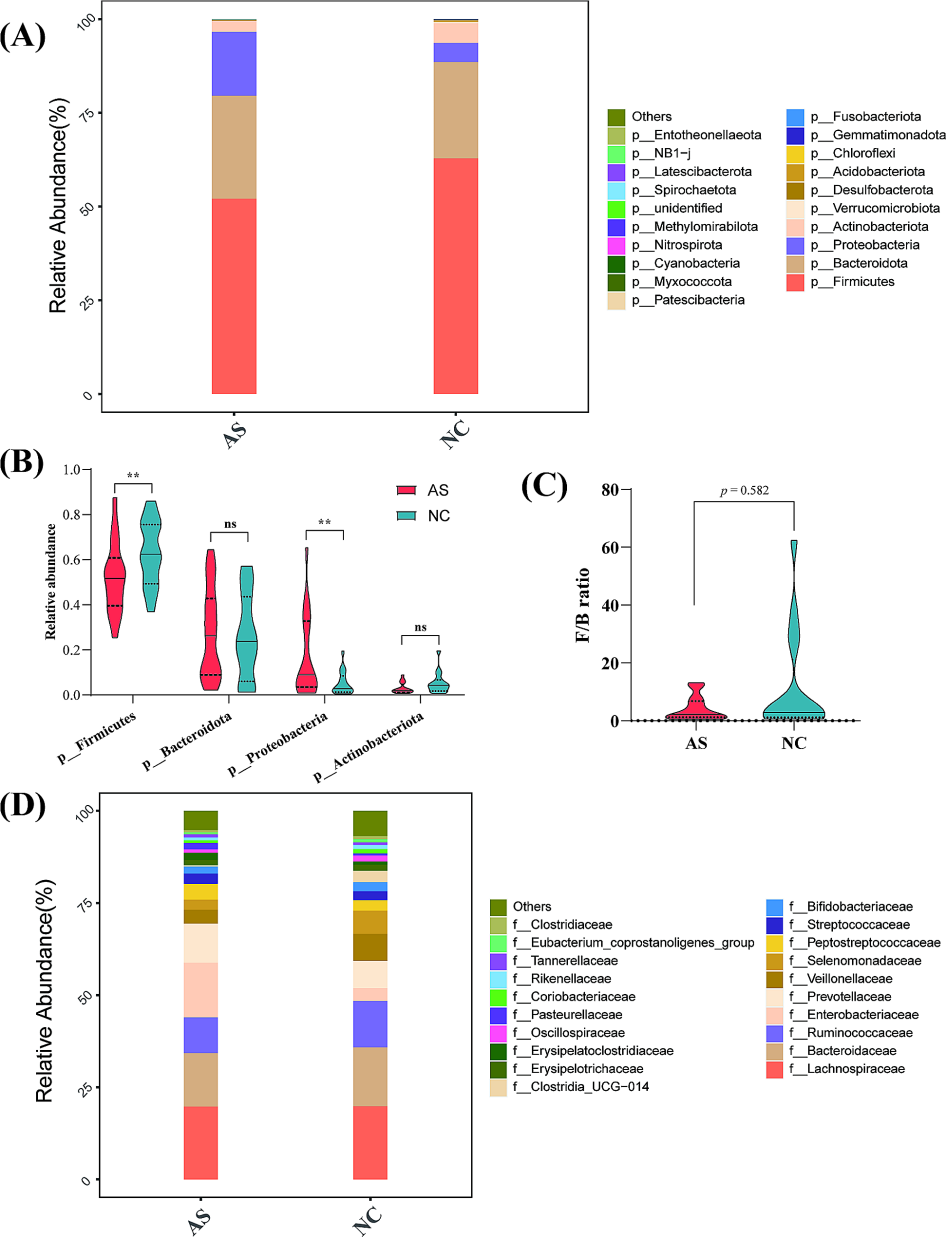
Taxonomic changes of the gut microbiota at the phylum and family levels. (**A**) relative abundance ratio of microbiota at the phylum level in two groups; (**B**) comparison in the relative abundance of Firmicutes, Bacteroidota, Proteobacteria, and Actinobacteria between the two groups; (**C**) the ratio of Firmicutes/Bacteroidota (F/B) between the AS group and NC group; (**D**) relative abundance ratio of microbiota at the family level in two groups

To further investigate gut microbial composition, we analyzed the results at the phylum level. *Firmicutes*, *Bacteroidota*, *Proteobacteria*, and *Actinobacteria* were the predominant phyla in both groups (Fig. 3A). Compared to the NC group, the relative abundance of *Firmicutes* was significantly decreased in the AS group (*p* = 0.042, Fig. 3B), whereas the relative abundance of *Proteobacteria* was significantly higher in the AS group (*p* = 0.016, Fig. 3B). No significant differences were observed in the relative abundance of *Bacteroidota* and *Actinobacteria* between the AS and NC groups (Fig. 3B). Furthermore, the ratio of *Firmicutes*/*Bacteroidota* (F/B) showed a lower trend in the AS group (Fig. 3C), although the difference was not significant (*p* = 0.582). At the family level, *Enterobacteriaceae*, *Erysipelatoclostridiaceae*, *Pasteurellaceae*, and *Lactobacillaceae* were predominant in the AS group, whereas *Erysipelotrichaceae* were prevalent in the NC group (Fig. 3D).

**Fig. 4:**
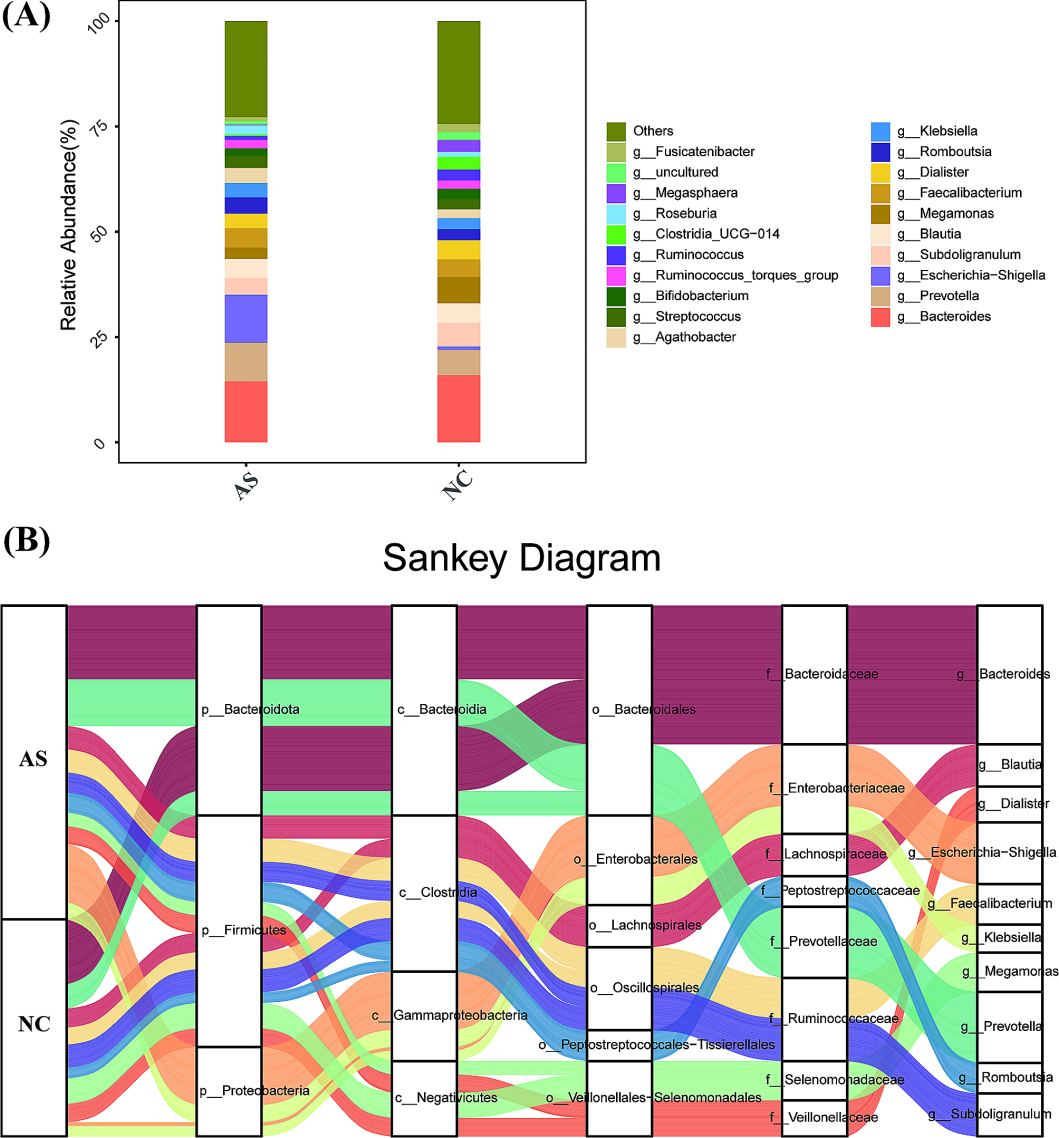
Taxonomic changes of the gut microbiota at the genus levels. (**A**) relative abundance ratio of microbiota at the genus level in two groups; (**B**) the Sankey diagram revealing the difference of microbial composition from the phylum level to the genus level

At the genus level, the gut microbiota composition was analyzed. Overall, the top five most abundant genera detected in the AS group were *Bacteroides* (14.47%), *Escherichia-Shigella* (11.43%), *Prevotella* (9.15%), *Blautia* (4.57%), and *Faecalibacterium* (4.56%), whereas those in the NC group were *Bacteroides* (15.95%), *Megamonas* (6.26%), *Prevotella* (5.98%), *Subdoligranulum* (5.60%), and *Blautia* (4.66%). Thus, the dominant genera in both group were *Bacteroides*, *Prevotella*, and *Blautia* (Fig. 4A). The Sankey diagram revealed the changing process of microbial composition from the phylum level to the genus level (Fig. 4B).

**Fig. 5:**
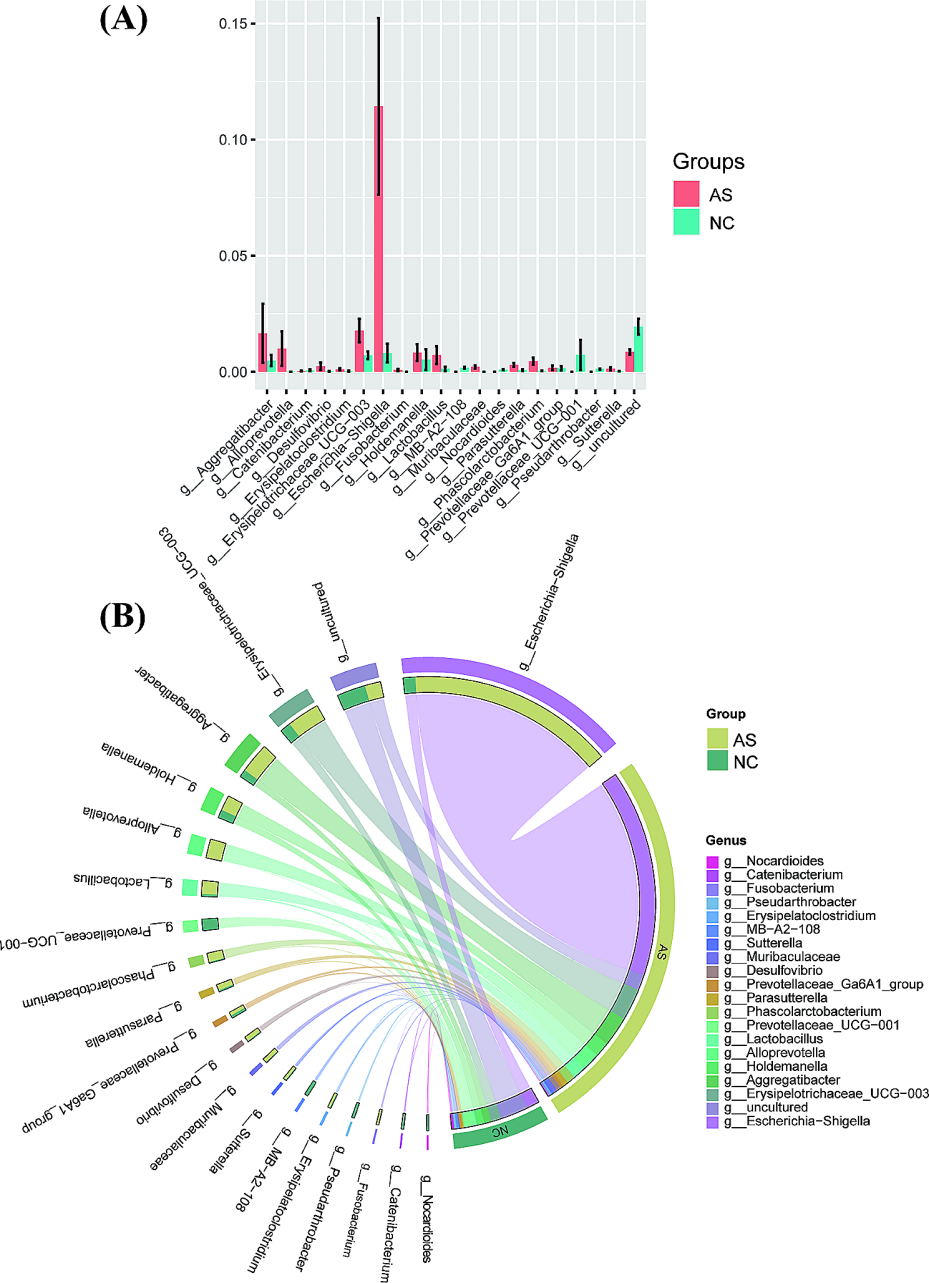
The top 20 relative abundance at the genus levels identified by the Wilcoxon rank-sum test. (**A**) the bar plot of comparing the top 20 relative abundance at the genus levels; (**B**) the Circos diagram revealing the relative abundances of the top 20 genera between two groups

To compare the taxonomic profiles between AS and NC, genera with relative abundance > 0.01% in each sample were selected. The Wilcoxon rank-sum test showed that fecal samples from the AS group exhibited a higher relative abundance of Escherichia-Shigella, *Erysipelotrichaceae_UCG-003*, *Aggregatibacter*, *Alloprevotella*, *Holdemanella*, *Lactobacillus*, *Phascolarctobacterium*, *Catenibacterium*, *Fusobacterium*, *Erysipelatoclostridium*, *Sutterella*, *Muribaculaceae*, *Desulfovibrio*, *Prevotellaceae_Ga6A1_group*, *Parasutterella*, and *Phascolarctobacterium*. The NC group showed a higher relative abundances of *Nocardioides*, *Pseudarthrobacter*, *MB-A2-108*, and *Prevotellaceae_UCG-001* (Fig. 5A). A Circos diagram showed the differences in the relative abundance of these genera between the two groups (Fig. 5B).

### Identifying key intestinal flora

**Fig. 6:**
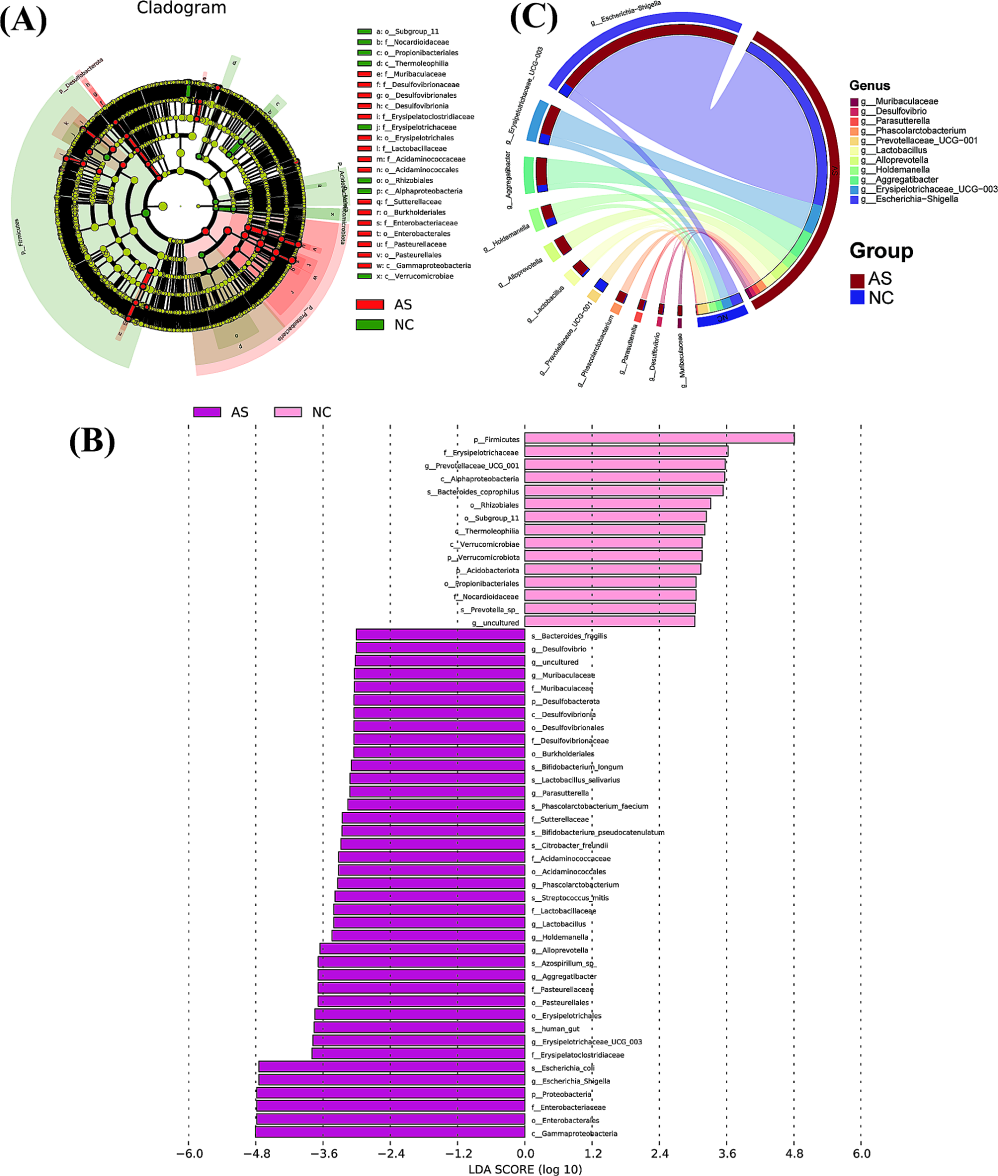
The differentially expressed gut microbiota identified by LEfSe analysis. (**A**) cladogram generated from LEfSe analysis showing the relationship between taxa; (**B**) LEfSe analysis (LDA score > 3) identified the taxa with the greatest differences in abundance between the two groups; (**C**) the Circos diagram revealing the relative abundances of 11 different genera identified by LEfSe analysis between two groups

Linear discriminant analysis effect size (LEfSe) identified differentially abundant taxa between the AS and NC groups. In total, 54 differentially expressed taxa were identified at a linear discriminant analysis (LDA) score > 3.0 (Fig. 6A and B). Of them, 39 were highly abundant in the AS group, whereas 15were highly abundant in the NC group. At the genus level, LEfSe feature selection identified notably higher abundances genera of *Escherichia_Shigella*, *Erysipelotrichaceae_UCG_003*, *Aggregatibacter*, *Alloprevotella*, *Holdemanella*, *Lactobacillus*, *Phascolarctobacterium*, *Parasutterella*, *Muribaculaceae*, and *Desulfovibrio* in the AS group, and enriched *Prevotellaceae_UCG_001* in the NC group (Fig. 6B). The Circos diagram showed the difference in the relative abundances of these identified genera between two groups (Fig. 6C). These results demonstrated specific changes in the gut microbial composition of the AS group compared to that of the NC group.

### Microbial co-occurrence network

**Fig. 7:**
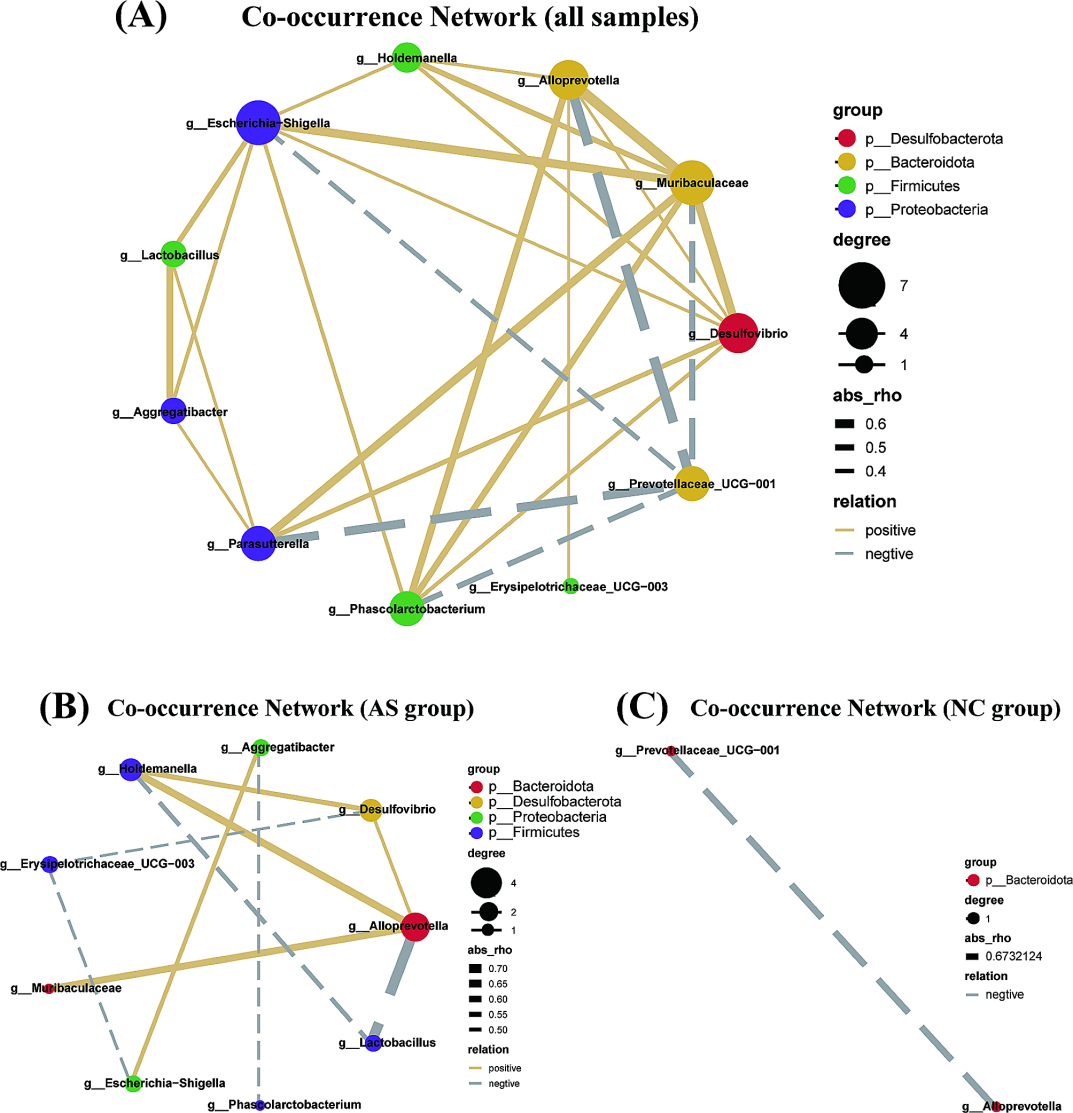
The co-occurrence network of the top 11 genera identified by the LEfSe analysis. (**A**) the co-occurrence network in all samples; (**B**) the co-occurrence network in the AS group; (**C**) the co-occurrence network in the NC group

The co-occurrence network of 11 key genera identified by the LEfSe analysis was constructed. These different genera were used to construct an interaction network presenting the relationships among intestinal flora markers (Spearman’s correlation, |correlation coefficient| > 0.3, *p* < 0.05). The co-occurrence network of all samples was mainly centered on *Escherichia_Shigella*, *Muribaculaceae*, and *Alloprevotella* (Fig. 7A). The genus of *Escherichia_Shigella* was positively correlated with all other key genera, respectively, except for *Prevotellaceae_UCG − 001*. The genus of *Prevotellaceae_UCG − 001* was negatively correlated with all other genera, respectively. Notably, AS-enriched genera (including *Escherichia_Shigella*, *Erysipelotrichaceae_UCG_003*, *Aggregatibacter*, *Alloprevotella*, *Holdemanella*, *Lactobacillus*, *Phascolarctobacterium*, *Muribaculaceae*, and *Desulfovibrio*, Fig. 7B) were more highly interconnected than NC-enriched genera (including *Prevotellaceae_UCG − 001* and *Alloprevotella*, Fig. 7C).

### Correlation analysis among key genera and clinical indicators

**Fig. 8:**
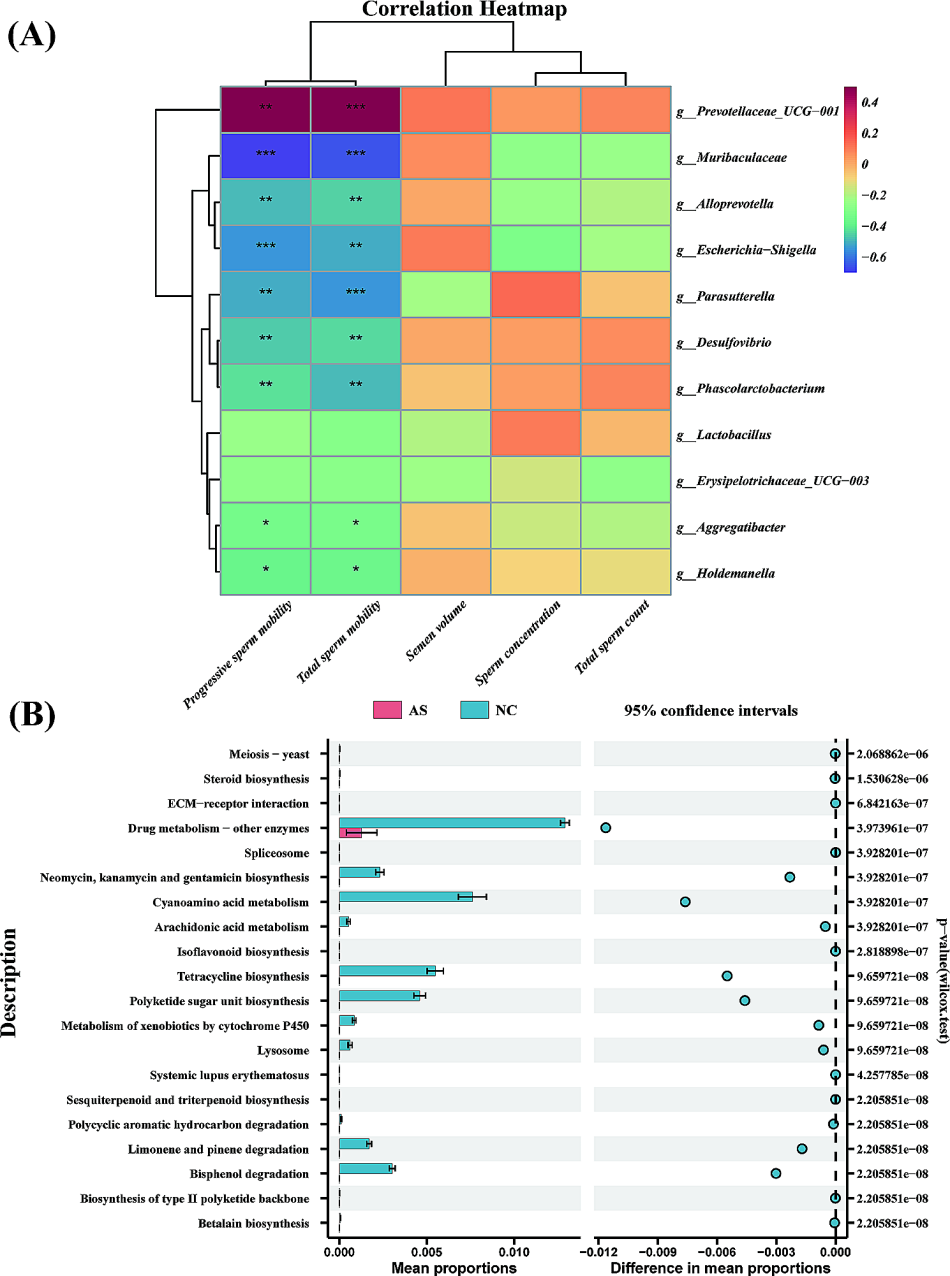
Correlation analysis and functional profile analysis of the gut microbiota. (**A**) the correlation heatmap showing the correlation outcomes among 11 differential genera (identified by the LEfSe analysis) and clinical sperm parameters; (**B**) Stamp analysis showing the different KEGG (level 3) pathways between two groups predicted by PICRUSt2

To explore the predictive and discriminatory power of intestinal flora in AS, we investigated the relationship between the relative abundances of the key genera (n = 11, identified by the LEfSe analysis) and clinical sperm parameters. Spearman’s correlation analysis was performed on the key genera and clinical sperm parameters. The results showed that the 11 key genera were all not correlated with semen volume, total sperm count, and sperm concentration (*p* > 0.05). The key genera of *Escherichia_Shigella*, *Erysipelotrichaceae_UCG_003*, *Aggregatibacter*, *Alloprevotella*, *Holdemanella*, *Lactobacillus*, *Phascolarctobacterium*, *Parasutterella*, *Muribaculaceae*, and *Desulfovibrio* were all negatively correlated with total sperm mobility and progressive sperm mobility, respectively. However, the key genera of *Prevotellaceae_UCG_001* was positively correlated with total sperm mobility and progressive sperm mobility, respectively. A correlation heatmap was generated to clearly show the above results (Fig. 8A).

### Functional profile analysis of the intestinal flora

PICRUSt2 was used to predict the different KEGG pathways and discuss the potential mechanisms of intestinal flora in the AS and NC groups. Of the 178 KEGG (level 3) pathways tested, 88 pathways were differentially enriched between the AS patients and NC men at the value of *p* < 0.05. Stamp analysis showed increased pathways in the AS group including selenocompound metabolism, sulfur metabolism, rna degradation, nitrogen metabolism, and purine metabolism. Meanwhile, the AS group exhibited reduced activity in some key pathways such as meiosis, drug metabolism, cyanoamino acid metabolism, tetracycline biosynthesis, polyketide sugar unit biosynthesis, and steroid biosynthesis (Fig. 8B).

## Discussion

In the present study, high-throughput sequencing technology was employed to examine the intestinal flora of AS patients and NC men. Our study demonstrated that intestinal flora characteristics in patients with AS were significantly different from that in normal males. A lower richness and diversity (α-diversity and β-diversity) of intestinal flora occurred in the AS patients. The relative abundance of *Proteobacteria*, which could lead to enteral inflammation and tumor, was significantly higher in AS men. Our research also identified key gut microbiota in AS patients, including 11 important genera such as *Escherichia_Shigella* and *Prevotellaceae_UCG_001*, which could serve as potential biomarkers for AS.

Recent research has found that an imbalance in microbiota diversity can result in many illnesses like non-alcoholic fatty liver disease [13]. The microbiota feature in human semen was previously analyzed and some relevant genera such as *Lactobacillus* and *Prevotella* were identified [14]. In our study, four significant phyla including *Proteobacteria*, *Firmicutes*, *Actinobacteria*, and *Bacteroidetes* were identified. Moreover, the major genera in the gut were also analyzed. The highest abundance in the genus level was *Bacteroides* in two groups. *Bacteroides* were regarded as a potentially harmful genus. Some prior animal studies demonstrated the existence of *Bacteroides* was negatively relevant to sperm concentration and motility [12, 15]. Moreover, we discovered a significant increase in the abundance of *Escherichia-Shigella*, *Erysipelotrichaceae_UCG-003*, *Aggregatibacter*, *Alloprevotella*, *Holdemanella*, *Lactobacillus*, along with a decrease in *Nocardioides*, *Pseudarthrobacter*, *MB-A2-108*, and *Prevotellaceae_UCG-001* in the gut of AS patients. *Escherichia-Shigella* is usually regarded as a harmful bacterium and could result in sepsis and hemorrhagic colitis. In some cases, *Escherichia-Shigella* could participate in the synthesis of genotoxin and was relevant to the defect in DNA duplication [16]. *Lactobacillus* is identified as a gram-positive bacterium and it could be associated with the synthesis of short-chain fatty acids. Even though some researchers reported short-chain fatty acids could be helpful for demic wellness, excessive *Lactobacillus* abundance in males could alter the semen pH and cause abnormal microenvironment of spermatogenesis. Dysbiosis of these key microbiotas could have a significant impact on sperm progressive mobility.

The co-occurrence network revealed that varying correlations within intestinal flora. In the network encompassing all samples, we found that *Prevotellaceae_UCG_001* was all negatively relevant to other genera. However, *Alloprevotella* and *Escherichia-Shigella* were identified as the core genera in the network encompassing AS samples. Meanwhile, we showed that the correlation richness decreased significantly from the AS group to the NC group. It indicated that the microbiota profile in AS patients altered, which could result in the alternation of host phenotype, including decreased sperm mobility. Besides, *Escherichia-Shigella* was identified as one of the most abundant genera in the AS group and was usually regarded as potentially noxious. *Escherichia-Shigella* was considerably relevant to other genera in the co-occurrence network. Therefore, we considered that these potentially harmful genera could exert synergistic effects on each other and contribute to the occurrence of AS.

PICRUSt2 software was employed to predict relevant metabolic pathways. Activity of the steroid biosynthesis pathway was significantly lower in the AS group than in the NC group. Sex hormones, which are a form of steroid, are well-known to play a role in semen quality. Prior research has shown that abnormalities in sex hormones can lead to impaired semen quality [17]. The alternation of sex hormone levels such as FSH, LH, and T levels is associated with testicular impairment, impeded sperm production and maturation, and reduced sperm motility [18]. The outcomes revealed a strong relationship between the intestinal flora and human metabolism. In addition, KEGG pathway analysis results showed a significant difference in glycerophospholipid metabolism between the two groups. Glycerophosphocholine and lysophosphatidylcholine were mainly produced by glycerophospholipid metabolism and these two metabolites were associated with semen quality [19, 20]. Therefore, the gut microbiota of AS men might affect sperm motility through abnormal metabolic activity.

As far as we know, the present study reported a strong association between gut microbiota alternation and AS development for the first time. The outcomes could may provide insight into innovative perceptions of the function of the gut microbiota in the occurrence of AS. Some limitations existed in our study and should be improved in future interventions. First, metabolites in serum or feces were not examined. The detection of metabolites and their changes to microbiota dysbiosis might offer some further insight regarding the detailed mechanism of AS. Second, metagenomics sequencing of gut microbiota might be useful in illustrating the molecular mechanisms of AS and providing guidance for the prevention, diagnosis, and management of AS. Nevertheless, our study also had some excellent advantages. First, the large sample size of our 16 S rDNA analysis lends credibility to our findings. Second, some factors such as inflammatory bowel disease and depression could potentially impact gut microbiota. Our study excluded patients with these comorbidities, which improved the accuracy of our findings. Third, our study innovatively analyzed and compared the dietary habits of the participants, which were also key factors influencing gut microbiota, and no significant differences were observed in dietary habits between the two groups. Thus, our findings were more convincing and valuable. In short, this research significantly contributes to the understanding of the pathogenesis and management of AS disease.

## Conclusion

It appears that the composition of intestinal flora in AS patients was different from those in healthy men, suggesting that AS development may be associated with intestinal flora dysbiosis. Key gut microbiota biomarkers were screened, and relevant metabolic pathways were predicted in our study. Gut microbiota had a potential role in discriminating AS patients from healthy controls and function as a promising biomarker of AS. These key gut microbiotas are expected to be applied to clinical studies in the future to provide a gut microbiome-based personalized approach for AS patients.

### Electronic supplementary material

Below is the link to the electronic supplementary material.

Supplementary File 1: The detailed analysis method of gut microbiota

## Data Availability

All data will be available from the corresponding author on reasonable request. The datasets generated and/or analyzed during the current study are available in the SRA database of NCBI .
