# Supplementary material for: Characteristics of gut microbiota in patients with asthenozoospermia: a Chinese pilot study
Source: BMC Microbiol. 2024 Jan 15;24:22. doi: 10.1186/s12866-023-03173-5 (PMC10789020; doi:10.1186/s12866-023-03173-5)
Supplement: Supplementary file 1 — The detailed analysis method of gut microbiota [file 12866_2023_3173_MOESM1_ESM.docx]

**Supplementary file 1. The detailed analysis method of gut microbiota**

**1. Feces specimen collection**

Fresh fecal samples were collected for gut microbiome analysis. Each man provided a single fecal sample. All fecal samples were collected using sterile containers and stored at −80 °C until DNA extraction.

**2. DNA extraction and quality checked**

Soil genomic DNA was extracted using E.Z.N.A. Soil DNA Kit (Omega Bio-tek, Inc., USA) following the manual. Concentration and quality of the genomic DNA were checked by NanoDrop 2000 spectrophotometer (Thermo Scientific Inc., USA). DNA samples were stored at - 20 ℃ for subsequent experiments.

**3. PCR amplification**

The V3-4 hypervariable region of bacterial 16S rRNA gene were amplified with the universal primer 338F (5’-ACTCCTACGGGAGGCAGCAG-3’) and 806R (5’- GGACTACNNGGGTATCTAAT-3’). For each sample, 8-digit barcode sequence was added to the 5’ end of the forward and reverse primers (provided by Allwegene Company, Beijing). The PCR was carried out on a Mastercycler Gradient (Eppendorf, Germany) using 25 μL reaction volumes, containing 12.5 μL 2× Taq PCR MasterMix (Vazyme Biotech Co., Ltd, China), 3 μL BSA(2ng/μL), 1 μL Forward Primer(5 μM), 1 μL Reverse Primer(5 μM), 2 μL template DNA, and 5.5 μL ddH_2_O. Cycling parameters were 95 ℃ for 5 min, followed by 28 cycles of 95 ℃ for 45 s, 55 ℃ for 50 s, and 72 ℃ for 45 s with a final extension at 72 ℃ for 10 min. The PCR products were purified using a Agencourt AMPure XP Kit (Beckman Coulter, Inc., USA). Sequencing libraries were generated using NEB Next Ultra II DNA Library Prep Kit (New England Biolabs, Inc., USA) following the manufacturer's recommendations. The library quality was assessed by Nanodrop 2000 (ThermoFisher Scientific, Inc., USA), Agilent 2100 Bioanalyzer (Agilent Technologies, Inc., USA), and ABI StepOnePlus Real Time PCR System (Applied Biosystems, Inc., USA), successively.

**4. High throughput sequencing**

Deep sequencing was performed on Illumina Miseq (Illumina, Inc., USA) platform. After the run, image analysis, base calling, and error estimation were performed using Illumina Analysis Pipeline Version 2.6 (Illumina, Inc., USA).

**5. Data analysis and processing**

The raw data was divided into different samples according to the barcode sequence through QIIME (v1.8.0) software. Pear (v0.9.6) software was used to filter and splice raw data. The sequences were removed from consideration if they were shorter than 120 bp, had a low-quality score (≤ 20), contained ambiguous bases. During splicing, the minimum overlap setting was 10bp, and the mismatch rate was 0.1. After splicing, VSEARCH (v2.7.1) software was used to remove sequences with length less than 230 bp and removed the chimeric sequence by UCHIME method according to the Gold Database. Qualified sequences were clustered into operational taxonomic units (OTUs) at a similarity threshold of 97% using uParse algorithm of VSEARCH (v2.7.1) software. To minimize the effect from sequencing depth to the inter-sample variation, samples were subsampled (rarefied) to 28304 sequences per sample by random sampling. The BLAST tool was used to classify all OTU representative sequences into different taxonomic groups against Silva138 Database, and e-value threshold was set to1e-5. QIIME (v1.8.0) was used to generate rarefaction curves and to calculate the richness and diversity indices based on the OTU information. Based on the results of taxonomic annotation and relative abundance, bar-plot diagram analysis was performed. To describe the dissimilarity between multiple samples, principal coordinates analysis (PCoA) was analyzed based on the OTU information from each sample. The β-Diversity distance matrix between samples were calculated using the unweighted UniFrac method. Linear discriminant effect size (LEfSe) analysis was performed with a LDA >3. The function of microbiota was predicted using PICRUSt2 (Phylogenetic Investigation of Communities by Reconstruction of Unobserved States) software. STAMP software screened functional differences between AS and NC groups.
